# Supplementary material for: A Bayesian generative neural network framework for epidemic inference problems
Source: Sci Rep. 2022 Nov 16;12:19673. doi: 10.1038/s41598-022-20898-x (PMC9667449; doi:10.1038/s41598-022-20898-x)
Supplement: Supplementary file 1 — Supplementary Information. [file 41598_2022_20898_MOESM1_ESM.pdf]

Supplementary Materials of: ”A Bayesian generative neural network framework for epidemic inference problems”

Indaco Biazzo[1*], Alfredo Braunstein[1,2,3], Luca Dall'Asta[1,2,3], and Fabio Mazza[1]

[1] Politecnico di Torino, Corso Duca degli Abruzzi 24, 10129, Torino, Italy,
[2] IIGM - Italian Institute for Genomic Medicine, c/o IRCSS, 10060 Candiolo, Torino, Italy
[3] Collegio Carlo Alberto, P.za Arbarello 8, 10122, Torino, Italy

[*]To whom correspondence should be addressed; E-mail: indaco.biazzo@polito.it

1 Learning procedures and regularization

The learning procedure involves the minimization of the Kullback-Leibler divergence, eq.9 in the main text, between the posterior probability and the variational autoregressive neural networks. More precisely, the expression of the posterior can be factorized as a product over nodes (individuals) and times, $p\left(\underline{\mathbf{x}}|\mathcal{O}\right) \propto \prod_i \Psi_i((\underline{x}_i, \underline{\mathbf{x}}_{\partial i})) = \prod_{i,t} \psi_i^t\left(x_i^t, \mathbf{x}_{\partial i}^{t-1}\right)$ where

$$\psi_i^t\left(x_i^t, \mathbf{x}_{\partial \mathbf{i}}^{\mathbf{t-1}}\right) = \begin{cases} p_i^t\left(x_i^{(t)}|\mathbf{x}_{\partial i}^{t-1}\right)\prod_{(i_r,t_r)=(i,t)} p_r(O_r^{t_r} \mid x_i^t) & t>0 \\ p_i(x_i^0)\prod_{(i_r,t_r)=(i,t)} p_r(O_r^{t_r} \mid x_i^t) & t=0 \end{cases} \tag{1}$$

Since the factor ψ_i^t can be zero for some epidemic configuration $\underline{\mathbf{x}}$, for example when they are forbidden by dynamical constraints, the gradient of the KL divergence, Eq.12 in the main text, can diverge preventing the application of gradient descent algorithms. In these cases, the diverging term $\log\psi_i^t = -\infty$ is replaced with a regularization term $\log\epsilon$ with $\epsilon \ll 1$. The

gradient of the KL divergence with respect to the parameters of the trial distribution reads

$$\nabla_\theta D_{KL}(q||p) = \nabla_\theta \sum_{\underline{x}} q_\theta\left(\underline{\mathbf{x}}\right)\left[-\log p\left(\underline{\mathbf{x}}|\mathcal{O}\right) + \log q_\theta\left(\underline{\mathbf{x}}\right)\right] \tag{2}$$

$$= \sum_{\underline{x}} \nabla_\theta q_\theta\left(\underline{\mathbf{x}}\right)\left[-\log\left(\prod_i \Psi_i\left(\underline{x}_i, \underline{\mathbf{x}}_{\partial i}\right)\right) + \log q_\theta\left(\underline{\mathbf{x}}\right)\right] + \sum_{\underline{x}} q_\theta\left(\underline{\mathbf{x}}\right)\nabla_\theta \log q_\theta\left(\underline{\mathbf{x}}\right) \tag{3}$$

$$= \sum_{\underline{x}} q_\theta\left(\underline{\mathbf{x}}\right)\left[-\log\left(\prod_i \Psi_i\left(\underline{x}_i, \underline{\mathbf{x}}_{\partial i}\right)\right) + \log q_\theta\left(\underline{\mathbf{x}}\right)\right]\nabla_\theta \log q_\theta\left(\underline{\mathbf{x}}\right), \tag{4}$$

where we used that $\sum_{\underline{x}} q_\theta\left(\underline{\mathbf{x}}\right)\nabla_\theta \log q_\theta\left(\underline{\mathbf{x}}\right) = \sum_{\underline{x}} \nabla_\theta q_\theta(\underline{\mathbf{x}}) = 0$ and $\log(Z)\sum_{\underline{x}} \nabla_\theta q_\theta(\underline{\mathbf{x}}) = 0$ due to the normalization $\sum_{\underline{x}} q_\theta(\underline{\mathbf{x}}) = 1$. The presence of large negatives values in the derivatives (e.g. due to $\log\epsilon$ terms) reduces the ability of gradient descent algorithms to explore all configurations compatible with the constraints. To overcome this issue, an annealing procedure is adopted, in which a fictitious inverse temperature β is introduced in the computation the gradient of the KL divergence,

$$\nabla_\theta D^\beta_{KL}(q|p) = \sum_{\underline{x}} q_\theta\left[-\beta\log\left(\prod_i \Psi_i\left(\underline{x}_i, \underline{\mathbf{x}}_{\partial i}\right)\right) + \log q_\theta\right]\nabla_\theta \log q_\theta. \tag{5}$$

The minimization procedure starts with $\beta = 0$, where all the configurations are allowed with uniform probability, then the parameter β is slowly increased until it reaches $\beta = 1$, at which the original expression of the loss function is recovered.

2 Neural network architecture

In the proposed approach, each conditional probability function $p_i(\underline{x}_i|\underline{\mathbf{x}}_{<i})$ has to be approximated with a neural network $q_i^{\theta_i}\left(\underline{x}_i|\underline{\mathbf{x}}_{<i}\right)$. For monotonic models such as the SIR epidemic model, the state-space representation of temporal trajectories as a sequence $\underline{x}_i \in \mathcal{X}^{T+1}$ of $T+1$ individual states is redundant, and it turns out to be more efficient to represent them by the time instant $t_i^I \in (0, T+1)$ at which individual i becomes infected and time instant $t_i^R \in (0, T+1)$ at which it recovers, the time $T+1$ corresponding to the case of no infection or recovery occurred

for i in the interval $[0, T]$. More precisely, the case $\left(t_i^I = T+1,\, t_i^R = T+1\right)$ corresponds to individual i being susceptible at every time of the epidemic process, and $\left(t_i^I \neq T+1,\, t_i^R = T+1\right)$ corresponds to individual i being infected but not recovering in the time interval $[0, T]$.

This parametrization reduces the state space that is explored by our method, decreasing the number of incorrect time trajectories generated (for instances, time trajectory with transitions from state I to S).

In order to compute the probability of a trajectory of a single individual, we employ two neural networks, one for the time of infection, and one for the recovery time. Each of these networks is given as input a subset of the time trajectories of individuals with lower sorting index π_i (see next section), and the one of the recovery time is also given as input the infection time of the same individual. The input of the networks are the infection and recover times of previous individuals, encoded in *one hot* scheme. Therefore, the (conditional) probability distributions represented by the networks are, for each individual i, $q_i^{\theta_{i,I}}\left(t_i^I \mid \left\{t_j^I, t_j^R\right\}_{\pi_j<\pi_i}\right)$ for the infection times, and $q_i^{\theta_{i,R}}\left(t_i^R \mid \left\{t_j^I, t_j^R\right\}_{\pi_j<\pi_i}, t_i^I\right)$ for the recovery times, where $\theta_{i,I}$ and $\theta_{i,R}$ are the weights of each network.

In our implementation, each neural network is a multi-layer perceptron (MLP) composed of three hidden layers plus one output layer; each layer is fully connected, and can be written as $\underline{L}_{k+1} = \sigma\left(\mathbf{W_k}\underline{L}_k + \underline{b}_k\right)$ where $\underline{L}_k$ is the input vector (output of layer k), $\mathbf{W_k} \in \theta$ is the matrix of the weights, $\underline{b}_k \in \theta$ is the bias vector, and σ is the activation function, which is *Relu* for the hidden internal layers and *Softmax* for the last layer. The width of each layer (the number of neurons) varies linearly from the input size of each network to the output size.

The size of the input and outputs of the neural networks used for each individual depends on the observations and the contact graph in the following way: a) the input size depends on the number of individuals with lower index that are considered (depending on the approximation made, see section 2.1) and b) the observations made on an individual restrict the phase space

of the possible instants of infection (for example, if we observe individual i is in the infected state at time t_O, this means that his/her infection time $t_i^I \leq t_O$ and the recovery time $t_i^R > t_O$). This last condition affects the size of the output of each network, but also the input size of the networks for the individuals with higher index, whose network depends on it: for example, if individual i is observed in state S at the final time (T) with certainty, this implies that the infection and recovery times are fixed, $t_i^I = T + 1$ and $t_i^R = T + 1$, and the network will always give the same value for them. Thus, in this case, for all individuals j with $\pi_j > \pi_i$, the dependency on i will effectively disappear.

2.1 Graph approximations

We now discuss the approximation to the dependency of the conditional probabilities for the individuals' trajectories. The probability of a whole epidemic cascade can be written in the following autoregressive expression:

$$q^{\theta}(\underline{\mathbf{x}}) = \prod_i q_i^{\theta_i} \left(\underline{x}_i | \underline{\mathbf{x}}_{<i}\right), \tag{6}$$

The dependencies in the factors $q_i^{\theta_i}(\underline{x}_i|\underline{\mathbf{x}}_{<i})$ can be reduced, in the case of acyclic contact networks, to only those corresponding to the next-nearest neighbors with lower index (we relegate the derivation to section 3), but in the general case of contact networks with cycles it is just an approximation, which we employ for all contact networks analyzed in the present work.

In order to check this approximation, we have run several tests on the **patient zero** problem (see main text for the definition), applied to the proximity random contact graphs. For this purpose, we train the ANN either (a) considering the full dependencies on all nodes with lower permutation index (we call this version *full graph*), (b) consider only the dependency on the first and second neighbors (called *next nearest neighbors* approximation) with lower permutation index, which is the approximation used in all the rest of this work, (c) considering only the first

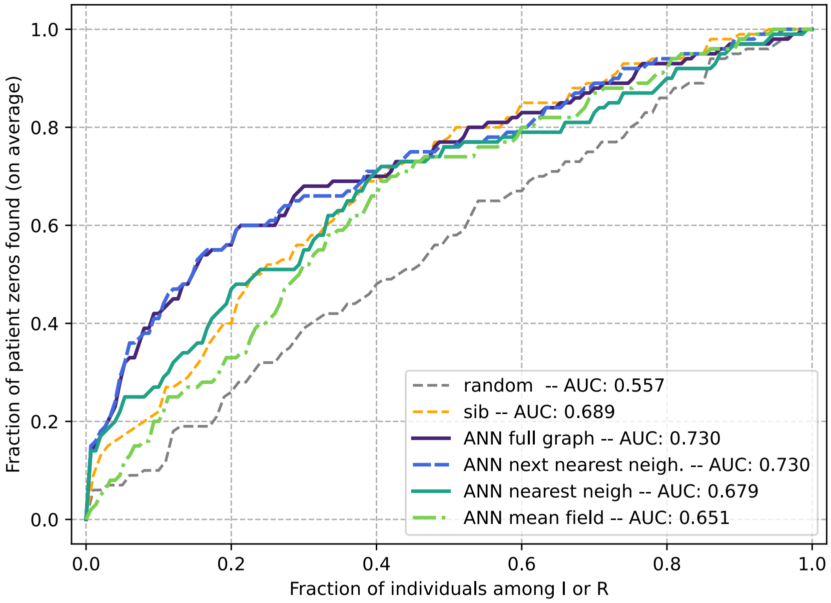

Figure 1: Accuracy in finding the patient zero with different approximations of the dependencies between individuals, compared with sib (Belief Propagation) method and random guessing of the source among the infected. The "full graph" case is when no approximation on the dependencies is made. The average is made over 100 epidemic cascade on contacts network generated from a **proximity** random graph (see main text) with $N = 100$ individuals. The epidemic parameters used fro the generation are $\lambda = 0.03$ and $\mu = 0.02$, with $T = 15$ time instants. The legend also shows the Area under each curve (AUC).

neighbors (*nearest neighbors*) with lower permutation index, or (d) ignoring the dependency on the rest of the graph (called *mean field* approximation).

Figure 1 shows how the accuracy in finding the patient zero is influenced by the approximation chosen, while in figure 2 the reduction of the number of parameters in the network, with respect to the full graph case, is shown. We see that the *next nearest neighbors* approximation gives estimates which are on par with the full graph case, while employing less parameters, thus reducing the space and time needed for the training.

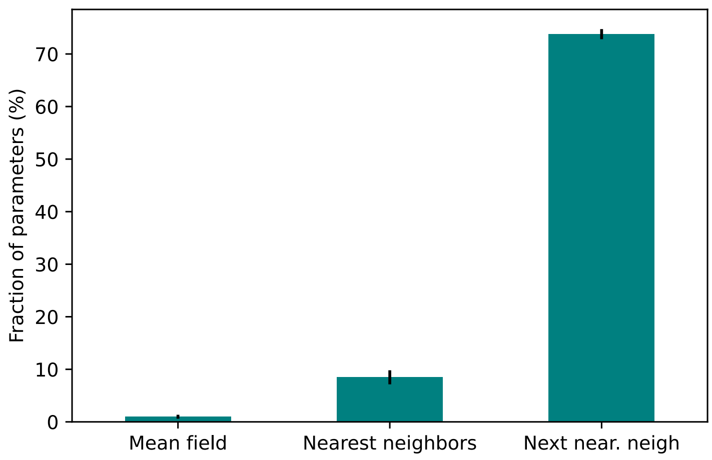

Figure 2: Percentage of parameters needed, on average, when considering the approximation of the dependencies between individuals, with respect to the case of no approximation as described in section 2.1. See figure 1 for the accuracy and details regarding the epidemic realizations.

2.2 Training procedure

To train the whole network, we do 10 000 steps of the annealing procedure described in the main text (except for the **hospital** case where we do 20 000 steps), increasing β linearly from 0 to 1. At each step, we generate 10 000 epidemic cascades before updating the weights using the ADAM optimizer [1] with learning rate $lr = 0.001$.

In the case of the risk inference problem, we also include a prior probability in the model, with strength decreasing linearly with β. This prior is computed from the probabilities of each individual being sampled as S, I or R at the last time instant $t = T$, and it is designed in such a way that, when $\beta = 0$, the probabilities for the three states are equal.

The running time of the ANN algorithm on a single instance ranges from one hour to two days on a single GPU (Nvidia TITAN RTX), depending on the structure of the observations $\mathcal{O}$ and that of the underlying contact network. Our implementation using the PyTorch framework [2] is publicly available in the repository [3]. The results presented in the main text and in SI can be reproduced using the following repository [4].

3 Simplified Conditional probability on acyclic graph

In this section we show that in the case of epidemic spreading in a acyclic interaction networks, it is possible to restrict the dependence of previous nodes in the conditional probabilities Eq.7, to the second neighbors with lower index. Let us consider the configuration x of N variables, a probability distribution $p(\underline{x}) = \frac{1}{Z}\prod_a \psi_a(x_a)$ factorized over a set of factors $\{a\}$. We first demonstrate the following statements:

Lemma 1 (Markov blanket)**.** *Let* $p(x) = \frac{1}{Z}\prod_a \psi_a(x_a)$*, and* $I \cup J \cup K = \{1,\ldots,N\}$ *be disjoint and assume no factor depends on* x_I *and* x_K *simultaneously. Then* $p(x_I, x_K|x_J) = p(x_I|x_J)p(x_K|x_J)$.

Proof. Considering proportionality $\propto$ with respect to x_I only $p(x_I|x_J,x_K) \propto p(x_I,x_J,x_K) \propto \prod_a \psi_a(x_a) \propto \prod_{a\in\partial I}\psi_a(x_a)$ and $p(x_I|x_J) \propto p(x_I,x_J) \propto \sum_{x_K}\prod_a \psi_a(x_a) \propto \prod_{a\in\partial I}\psi_a(x_a)$.As both distributions are normalized wrt x_I, they must be equal. This implies that

$$\begin{aligned} p(x_I,x_K|x_J) &= p(x_I,x_J,x_K)\,p(x_J)^{-1} \\ &= p(x_I|x_J,x_K)\,p(x_J,x_K)\,p(x_J)^{-1} \\ &= p(x_I|x_J)\,p(x_K|x_J) \end{aligned}$$

where the last line follows from the derivation above.

Lemma 2 (Separated neighborhood)**.** *Let* $p(x) = \frac{1}{Z}\prod_a \psi_a(x_a)$*, and* $G = (V \cup A, E)$ *be the associated bipartite factor graph, with* $E = \{(i,a) \in V \times A : \psi_a$ *depends on variable* $x_i\}$*. Let* $I \cup J \cup K \subseteq \{1,\ldots,N\}$ *be disjoint and assume that every path from* I *to* K *in* G *must pass through* J *(equivalently, removing vertices in* J *leave* I *and* K *separated). Then* $p(x_I,x_K|x_J) = p(x_I|x_J)p(x_K|x_J)$*, and* $p(x_I|x_{J\cup K}) = p(x_I|x_J)$

Proof. Let I' be the connected component of I in $G\setminus J$ and $K' = V\setminus(I'\cup J)$. As all paths from I to K pass through J, no factors can depend on variables both in I' and in K'. By Lemma 1,

$p(x_{I'}, x_{K'}|x_J) = p(x_{I'}|x_J)p(x_{K'}|x_J)$. Then $p(x_I, x_K|x_J) = \sum_{x_{I'\setminus I}} \sum_{x_{K'\setminus K}} p(x_{I'}, x_{K'}|x_J) = \sum_{x_{I'\setminus I}} p(x_{I'}|x_J) \sum_{x_{K'\setminus K}} p(x_{K'}|x_J) = p(x_I|x_J)p(x_K|x_J)$. Then $p(x_I|x_K, x_J) = p(x_I, x_K|x_J)p(x_K|x_J)^{-1} = p(x_I|x_J)$.

Corollary 1 (Restricted autoregression)**.** *By calling $I = \{i\}$, $J = \{j < i : j \in \partial i\}$ and $K = \{j < i : j \notin \partial i\}$, we obtain that for an ordering of nodes such that J separates i from K we get $p(x_i|\{x_j : j \in \partial i, j < i\}) = p(x_i|\{x_j : j < i\})$.*

The last corollary is defined for a single node i. In considering the problem of approximating the posterior probability of an epidemic spreading process we distinguish two graphs: the first one, the *contact* graph, the nodes are the time trajectory of the states $\underline{x_i}$ of each individuals and the edges are the contacts between them. The second, the *factor* graph, has as nodes, again, the time trajectory of individuals and as factors those ($\{\Psi_i\}$) in the equation 6 in the main text. If the *contact* graph is acyclic and the nodes are topological ordered[5] then, thanks to the corollary 1, the following identity holds:

$$P(\underline{\mathbf{x}}|\mathcal{O}) = \prod_i P(\underline{x}_i|\mathbf{x}_{<i}) = \prod_i P(\underline{x}_i|\underline{\mathbf{x}}_{\partial^2<i}) \tag{7}$$

where $\mathbf{x}_{\partial^2<i}$ define the sets of nodes up to the next nearest neighbors (in the *contacts* graph) with index lower than i. The set of nodes $\mathbf{x}_{\partial^2<i}$ in the *contact* graph correspond to the $\mathbf{x}_{\partial<i}$ in the *factor* graph. To better visualize the two graphs, in Fig.3 we show two cases of *contact* acyclic graphs. The first one represents a linear chain defined by the contacts among individuals. The corresponding *contact* graph is acyclic but the *factor* graph contains cycles. In this case, for instance, considering the conditional probability of node 7 we have that $P(\underline{x}_7|\underline{\mathbf{x}}_{<i}) = P(\underline{x}_7|\underline{\mathbf{x}}_{\partial^2<i}) = P(\underline{x}_7|\underline{x}_4, \underline{x}_6)$. The node $4, 6$ separated the nodes 7 from the previous nodes in the *factor* graph, as the corollary 1 require. Similarly, the same concept apply to the second case, where we consider a tree graph. In both cases the nodes are ordered topologically so the equation (7) holds for each conditional probability.

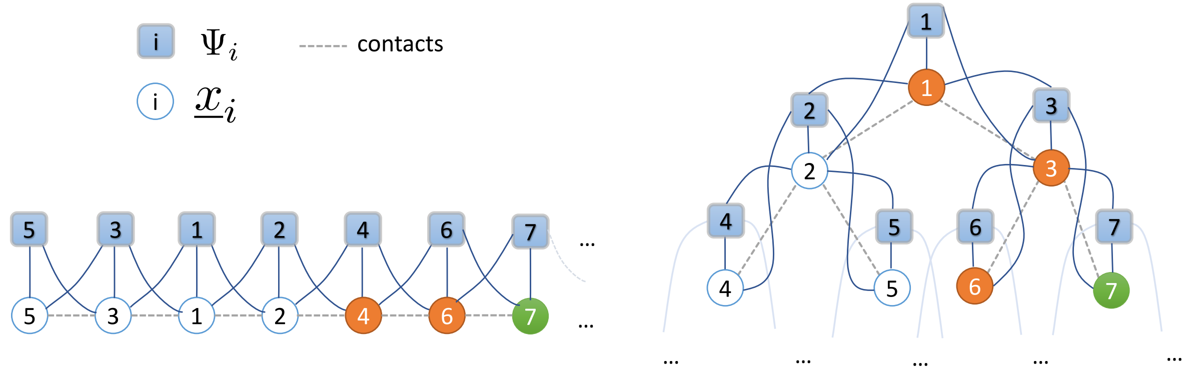

Figure 3: Two example of acyclic contact graphs ordered topologically. The two cases represent a linear chain on the left, and a tree on the right. The square represents the factor Ψ_i of the posterior probability of the dynamical process we want approximate. The circle represents the time trajectory of the states $\underline{x}_i$ of each individuals. The dashed lines are the contacts among individuals, and in both cases they generate a acyclic *contact* graph. Instead For instance the nodes 7 is separated (in the *factor* graph) from the rest of graph containing the nodes with minor index by the orange nodes.

4 Robustness of results with respect to the epidemic model

The main text shows results for different classes of epidemic inference problems. These problems depend on several parameters that could change the scenario analyzed. For instance, in the case of patient zero problems, we can vary the time T of the observed snapshot, the number of individuals, or the degree of the graphs in the case of synthetic interaction networks or the epidemic like the infectious or recovery parameters. All cases analyzed show strong fluctuations, imposing to average over a large number of instances to have a proper statistical significance of the results and appreciate the difference in the performance of the methods compared. As specified in the main text, we chose the infectious and recover parameters from having, on average, half of the infected individuals to decrease the instances where very few or almost all of them are infected (see fig.4, right plot). In these cases, the inference problems analyzed become

trivial or impossible to solve. On the other hand, our method has a computational cost that spans several hours on modern GPU to reach convergence for each instance analyzed, limiting the possibility of exploring the large dimensional space of the parameters of our inference problems. Nevertheless, we check how the performances of the methods analyzed vary, in the patient zero and the inference of parameters problems, with respect to the infectious parameters (λ or γ) to check the robustness of the results shown in the main text.

For the patient zero inference problem, we consider the **work** contact graph, and look at the performance of the different algorithms used, changing the infectivity γ. The figure 4 shows the area under the curves representing the fraction of times the patient zero is found, as in Figure 4 of the main text (right plots). The ANN method shows consistent performance with changing γ, giving the best score or on par with others algorithms.

Then we check the robustness of the inference of parameters in the case of of **rrg** interaction graph; the figure 5 shows that the inference of the infectivity parameter λ remains good as λ varies.

5 Soft margin estimator

We also use the Soft Margin estimator from [6] for finding the patient zero. This is a Monte Carlo based estimator, which applies Bayes formula to estimate the source probability $\mathbb{P}\left(s=i\right)$: given an epidemic $\mathbf{x}(i)$ which is the result of the simulation, starting from individual i, and the set of observation $\mathbf{x}_O$ on the epidemic, the probability of node k being the source of the epidemic is

$$\mathbb{P}\left(s=k \mid \mathbf{x}_O\right) \propto P\left(\mathbf{x}_O \mid s=k\right) P\left(i\right) \tag{8}$$

We have extended the method to the SIR model. To evaluate $P\left(\mathbf{x}_O \mid s=k\right)$, we run M Monte Carlo simulations in which the source of the epidemic is k and compare the resulting epidemic cascade $\mathbf{x}_i$ with the observations. For this we use the Jaccard similarity function $\phi_X\left(\mathbf{x}_i, \mathbf{x}_O\right)$,

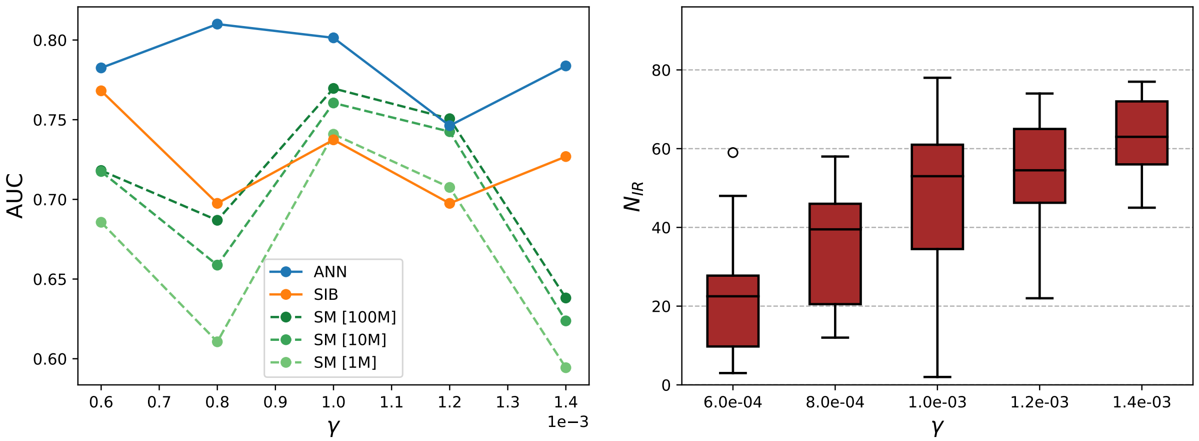

Figure 4: Accuracy in finding the patient zero while varying infectivity on the **work** contact graph. Here we look at the performance by the area under the curve (AUC) of the fraction of patient zeros found as a function of the fraction of considered individuals. We consider 20 different epidemic realizations for the γ values $\gamma = 0.6 \cdot 10^{-3}$, $\gamma = 0.8 \cdot 10^{-3}$, $\gamma = 1.2 \cdot 10^{-3}$, $\gamma = 1.4 \cdot 10^{-3}$, and 100 realizations for $\gamma = 10^{-3}$, while the recovery rate is fixed $\mu = 0.02$. On the right pane, the boxplot shows the number of infected individuals with the different values of γ, in a total population of 95 individuals.

relating how many individuals are in state X (either infected, $X = I$ or recovered, $X = R$) in the generated cascade and the observations. Clearly, if no individuals are observed in state X, then $\phi_X = 1$ regardless of the realization. The probability of observing a certain configuration, given the source of the epidemic, can then be written as:

$$P\left(\mathbf{x}_O \mid s = k\right) = \frac{1}{M} \sum_{i=1}^{M} \exp\left\{-\frac{\left(1 - \phi_I\left(\mathbf{x}_i, \mathbf{x}_O\right)\right)^2 + \left(1 - \phi_R\left(\mathbf{x}_i, \mathbf{x}_O\right)\right)^2}{a^2}\right\} \tag{9}$$

where the coefficient a regulates the sharpness of the peak around the case of perfect matching of observations ($\phi_I = 1$ and $\phi_R = 1$), where the corresponding value in the sum is 1. For the patient zero problems, we select the alpha values that give better results (retrospectively) because they seem to be strongly dependent on the contact graphs and the number of samples considered.

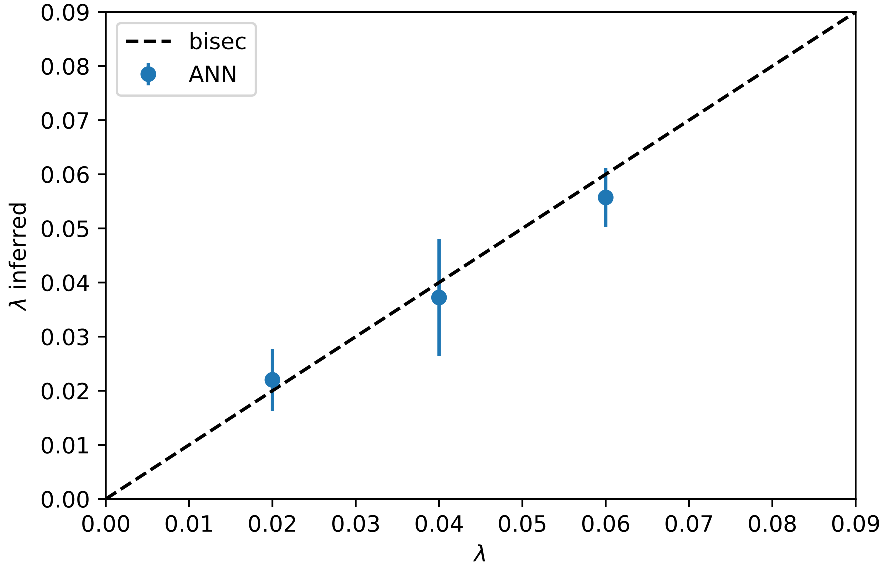

Figure 5: Inference of infectiousness parameter λ in the **rrg** case (100 individuals interacting according a random regular graph with degree 10 for 15 days). The problem setting is equals to that explained in the subsection ”Epidemic Parameters inference” in the main text. The plot shows the infectious parameter λ inferred by ANN with the perfect inference line $\lambda_{est} = \lambda$ highlighted. ANN correctly estimated them in error margin.

References

[1] Diederik P. Kingma and Jimmy Ba. Adam: A Method for Stochastic Optimization. *arXiv:1412.6980 [cs]*, January 2017. URL `http://arxiv.org/abs/1412.6980`. arXiv: 1412.6980.

[2] Adam Paszke, Sam Gross, Francisco Massa, Adam Lerer, James Bradbury, Gregory Chanan, Trevor Killeen, Zeming Lin, Natalia Gimelshein, Luca Antiga, Alban Desmaison, Andreas Kopf, Edward Yang, Zachary DeVito, Martin Raison, Alykhan Tejani, Sasank Chilamkurthy, Benoit Steiner, Lu Fang, Junjie Bai, and Soumith Chintala. Pytorch: An imperative style, high-performance deep learning library. In H. Wallach, H. Larochelle, A. Beygelzimer, F. d'Alché-Buc, E. Fox, and R. Garnett, editors, *Advances in Neural*

Information Processing Systems 32, pages 8024–8035. Curran Associates, Inc., 2019. URL http://papers.neurips.cc/paper/9015-pytorch-an-imperative-style-high-per

[3] Indaco Biazzo and Fabio Mazza. annfore: [a]utoregressive [n]eural [n]etworks [for] [e]pidemics inference problems. *code-repository*. doi: https://zenodo.org/badge/latestdoi/405138309. URL https://github.com/ocadni/annfore.

[4] Indaco Biazzo and Fabio Mazza. Repository of results for autoregressive neural network for epidemics. *code-repository*, 2022. doi: https://zenodo.org/badge/latestdoi/405138833. URL https://github.com/ocadni/annfore-results.

[5] Donald Knuth. *The art of computer programming*. Addison-Wesley, Reading, Mass, 1997. ISBN 0-201-89683-4.

[6] Nino Antulov-Fantulin, Alen Lančić, Tomislav Šmuc, Hrvoje Štefančić, and Mile Šikić. Identification of Patient Zero in Static and Temporal Networks: Robustness and Limitations. *Physical Review Letters*, 114 (24):248701, June 2015. doi: 10.1103/PhysRevLett.114.248701. URL https://link.aps.org/doi/10.1103/PhysRevLett.114.248701. Publisher: American Physical Society.
